# Galcanezumab effects on incidence of headache after occurrence of triggers, premonitory symptoms, and aura in responders, non-responders, super-responders, and super non-responders

**DOI:** 10.1186/s10194-023-01560-x

**Published:** 2023-03-16

**Authors:** Sait Ashina, Agustin Melo-Carrillo, Ajayi Toluwanimi, Nicolas Bolo, Edina Szabo, David Borsook, Rami Burstein

**Affiliations:** 1 Department of Anesthesia, Critical Care and Pain Medicine, Beth Israel Deaconess Medical Center, Boston, MA USA; 2 Department of Anesthesia, Harvard Medical School, Boston, MA USA; 3 Comprehensive Headache Center, Beth Israel Deaconess Medical Center, Boston, MA USA; 4 Department of Neurology, Beth Israel Deaconess Medical Center, Boston, MA USA; 5 Clinical Research Center, Beth Israel Deaconess Medical Boston, Boston, MA USA; 6 Departments of Psychiatry, Beth Israel Deaconess Medical Center, Harvard Medical School, Boston, MA USA; 7 Departments of Psychiatry, Massachusetts General Hospital, Harvard Medical School, Boston, MA USA; 8 Departments of Radiology, Massachusetts General Hospital, Harvard Medical School, Boston, MA USA; 9 Center for Life Science, Room 649, 3 Blackfan Circle, Boston, MA 02215 USA

**Keywords:** Migraine, Trigeminal, Hypothalamus, CGRP monoclonal antibodies, Central sensitization

## Abstract

**Background:**

The goal of this observational, open-label, cohort study was to determine whether prophylactic migraine treatment with galcanezumab, a peripherally acting drug, alters the incidence of premonitory symptoms, and/or occurrence of headache after exposure to triggers or aura episodes in treatment-responders (≥ 50% reduction in monthly migraine days [MMD]), super-responders (≥ 70%), non-responders (< 50%) and super non-responders (< 30%).

**Methods:**

Participants were administered electronic daily headache diaries to document migraine days and associated symptoms one month before and during the three months of treatment. Questionnaires were used to identify conscious prodromal and trigger events that were followed by headache prior to vs. after 3 months of treatment.

**Results:**

After 3 months of galcanezumab treatment, (a) the incidence of premonitory symptoms that were followed by headache decreased by 48% in the 27 responders vs. 28% in the 19 non-responders, and by 50% in the 11 super-responders vs. 12% in the 8 super non-responders; (b) the incidence of visual and sensory aura that were followed by headache was reduced in responders, non-responders, and super-responders, but not in super non-responders; (c) the number of triggers followed by headache decreased by 38% in responders vs. 13% in non-responders, and by 31% in super-responders vs. 4% in super non-responders; and (d) some premonitory symptoms (e.g., cognitive impairment, irritability, fatigue) and triggers (e.g., stress, sleeping too little, bright light, aura) were followed by headache only in super non-responders.

**Conclusions:**

Mechanistically, these findings suggest that even a mild decrease in migraine frequency is sufficient to partially reverse the excitability and responsivity of neurons involved in the generation of certain triggers and potentially premonitory symptoms of migraine.

**Trial registration:**

ClinicalTrials.gov: NCT04271202. Registration date: February 10, 2020.

## Background

The premonitory phase of migraine is commonly defined as a phase in which symptoms appear that correctly predict the onset of the headache phase [1]. Most predictive among such symptoms are enhanced sensitivity to light and noise, neck stiffness and pain, fatigue, decreased cognitive functions, and a wide range of negative emotions (irritability, annoyance, tension, anxiousness, depression) [2–6]. While some premonitory symptoms appear as early as 3 days prior to the onset of headache, quantitative data and diligent use of e-diaries revealed that in the majority of patients, subjective premonitory symptoms appear in the 0–12 h that precede the onset of headache [7]. As these symptoms can present themselves at any given time and for no apparent reason, it is not known why they occur and how they end up activating meningeal nociceptors and/or give rise to the very unique characteristics of migraine headache (e.g., often unilateral, often periorbital, often behind the eye, often throbbing). However, with the emergence of neuroimaging, it became apparent that groups of neurons and neural circuits that are in functionally related brain regions that may mediate premonitory symptoms of migraine are hypersensitive, hyperresponsive and hyperexcitable during the interictal phase of migraine [8–17]. These include neurons in multiple cortical, subcortical, diencephalic and brainstem regions that constitute the default mode network, salience network, frontoparietal network, executive network, and sensorimotor network [14, 18–28]. The overwhelming evidence for abnormal neuronal excitability in migraine patients has raised the notion that because the migraine brain is hyperexcitable, symptoms such as yawning, food craving, irritability, and feeling fatigue—which are common in humans—are followed by headache only in people with migraine but not in people without migraine. Along this line, it is also plausible that because of the migraine brain, environmental stimuli such as exposure to bright light [29], loud noise [30], certain odors [31, 32], cold or warm air, and changing barometric pressure [33] or small deviation from physiological and emotional homeostasis caused by skipping meals, insufficient sleep [34], letdown after stress [35], and menses [36] can trigger a headache in people with but not without migraine. While this explanation is generally acceptable, it is not fully understood why and how the migraine brain becomes hyperexcitable. The two most commonly discussed explanations include genetic susceptibility [37–40] and the possibility that the continuous arrival of nociceptive signals at brainstem, hypothalamic, thalamic, cortical and subcortical areas alter physiological, cellular and molecular properties of neurons involved in migraine premonitory symptoms and triggers [35, 41–43]. To challenge the second, and far less explored option, we sought to determine (a) whether the incidence of premonitory symptoms that are followed by headache decreases or remained unchanged in patients whose migraine frequency is reduced by galcanezumab, a migraine prophylactic anti-calcitonin gene related peptide (CGRP) monoclonal antibody that is approved in the US for the preventive treatment of migraine in adults, and (b) whether a prolonged period of reduced migraine frequency decreases patients’ vulnerability to common migraine triggers. This thesis is consistent with conscious and/or unconscious nociceptive inputs (including silent nociceptors [44]) that produce alterations in brain responsivity in different pain conditions [45] including migraine.

## Methods

The purpose of this observational, open-label, cohort study was to determine the impact of galcanezumab on migraine premonitory symptoms and triggers in treatment responders and treatment non-responders. The study was approved by local institutional review board (CCI Protocol # 2019P001081) and was conducted according to Good Clinical Practice and the Declaration of Helsinki. Patients provided written informed consent before participating in the study.

### Study participants

Study participants were recruited at the BIDMC Comprehensive Headache Center, Boston, MA.

Inclusion criteria for the study were between the ages of 18 and 65 years, previous diagnosis of migraine (with or without aura) in accordance with the diagnostic criteria of third edition of the International Classification of Headache Disorders—3 (ICHD-3) criteria [46], experience of headache ≥ 8 days per month (based on participants’ recalling their experience during the last 3 months), and onset of migraine at age 50 years or younger. Participating patients agreed to refrain from initiating or changing the type, dosage, or frequency of any prophylactic medications for indications other than migraine that may interfere with the study objectives (e.g., antidepressants, anticonvulsants, beta-adrenergic blockers, etc.). Exclusion criteria were current regimen of 1 or more migraine preventative therapy, pregnancy, breastfeeding, significant psychiatric or cognitive disorder and/or behavioral problems that could interfere with the study, other significant pain problem (e.g., cancer pain, fibromyalgia, other head or facial pain disorder), known or suspected severe cardiac disease (e.g., symptomatic coronary artery disease, prior myocardial infarction, congestive heart failure), known or suspected cerebrovascular disease (e.g., prior stroke or transient ischemic attack, symptomatic carotid artery disease, prior carotid endarterectomy or other vascular neck surgery), report of abnormal electrocardiogram within the last year (e.g., second- or third-degree heart block, prolonged QT interval, atrial fibrillation, atrial flutter, history of ventricular tachycardia or ventricular fibrillation, clinically significant premature ventricular contraction), uncontrolled high blood pressure (systolic > 160 mmHg, diastolic > 100 mmHg), known history or suspicion of secondary headache, known history or suspicion of substance abuse or addiction (within the last 5 years), current use of marijuana (including medical marijuana) or has used marijuana (including medical marijuana) or cannabidiol oil within the last 1 year. Other exclusion criteria were current use of simple analgesics or NSAIDs > 15 days per month or triptans, ergots, or combined analgesics > 10 days per month for headaches or other body pain. Patient could not participate if they currently used opioids for headaches or other body pain, underwent nerve block (occipital or other) in the head or neck within the last 3 months, or received botulinum toxin or anti-CGRP monoclonal antibody treatment within the last 6 months.

### Study design, electronic daily headache diary and interviews

**Fig. 1:**
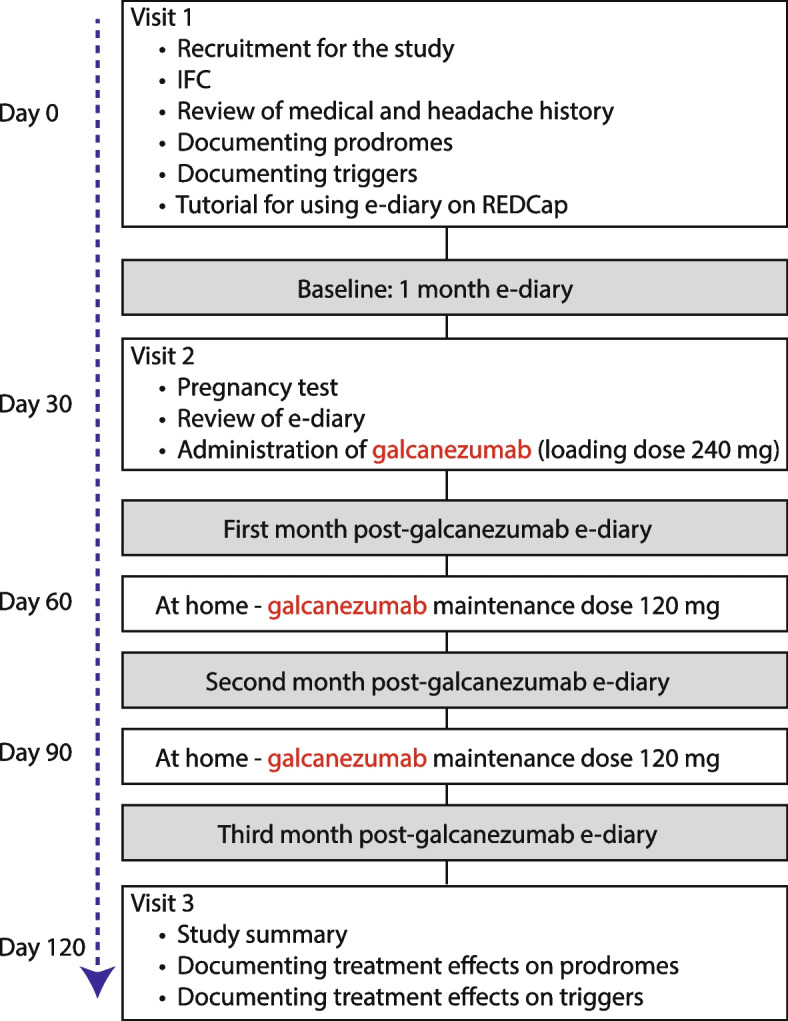
Study flowchart

Study participants were scheduled for 3 visits at the Headache Center (Fig. 1). In the first visit, selected candidates (i.e., those who cleared all inclusion/exclusion criteria) received a thorough explanation regarding the goals of the study and their role. Those who chose to participate signed an informed consent and were then asked to fill a headache questionnaire that included demographics, disease duration, family history, location of headache, frequency of attacks, associated symptoms (symptoms that appear during but not before onset on headache), premonitory symptoms (symptoms that appear before onset of headache, including aura), triggers, list of headache medications, and comorbid conditions. To document treatment effects on premonitory symptoms and triggers, participants received a detailed explanation about the differences between them. Briefly, a premonitory symptom was defined as an early sign or symptom that often indicates onset of a headache attack. Participants were asked to mark all relevant premonitory symptoms from the following list: nausea, vomiting, abdominal pain, diarrhea, increased appetite, decreased appetite, constipation, increased urination, tiredness, yawning, eye tearing, nasal congestion, dizziness, vertigo, ringing in the ear, nervousness/anxiousness, irritability, confusion, difficulty with concentration, difficulty with memory, feeling sad and depressed, light sensitivity, sound sensitivity, odor sensitivity, visual aura, sensory aura, speech aura, neck stiffness, jaw pain. Defining a migraine trigger as an endogenous or exogenous event that lowers the threshold for the onset of the headache phase of migraine, participants were asked to mark all relevant triggers from the following list: menses, stress, letdown after stress, skipping a meal, not drinking enough, sleeping too little/much, being too hot/cold, bright light, loud noise, certain smell, weather changes, air travel, food and wine. Participants were then asked to mark those premonitory symptoms they recognized as appearing before onset of migraine headache and those triggers they recognized as likely to be followed by migraine headache. Before leaving, they received a detailed tutorial on how to use the electronic daily headache diary on REDCap (see below).

In the second visit, after patients had completed a 1-month electronic daily headache diary, female participants went to the clinical research center for their pregnancy test and then to the headache center to receive the first dose of galcanezumab. During this visit they also learned how to use the injectors at home. Those uncomfortable injecting themselves at home were booked to receive the 2^nd^ and 3^rd^ injections at the headache center.

In the third visit, after participants had completed the electronic daily headache diary during the 3-month treatment period, they were given the same list of premonitory symptoms and triggers they completed before treatment initiation and were asked to mark those premonitory symptoms that continued to precede the onset of headache and those triggers that continued to cause their headache during the third month of treatment. They were also asked to mark premonitory symptoms that occurred but were not followed by headache during this period and those triggers that no longer caused them to have a headache. Premonitory symptoms and triggers were marked as followed by headache if they happened at least once during the last month of treatment. Only those premonitory symptoms and triggers that occurred but were not followed by a single headache were marked as no longer causing (i.e., triggers) the headache or being related (premonitory) to it.

### Electronic daily headache diary

All participants completed an electronic daily headache diary for at least 4 months (1 month before and 3 months after treatment initiation (Fig. 1). The electronic daily headache diary was administered in the form of a REDCap survey using an email link that participants accessed from their personal computer/electronic device. It consisted of a questionnaire that helped us determine daily/monthly incidence of headache and migraine, laterality (unilateral, bilateral), pain intensity (0–10 visual analogue scale), and daily occurrence of symptoms necessary to determine whether their headache was a migraine (e.g., nausea, vomiting, throbbing, photophobia, phonophobia, osmophobia).

### Treatment protocol

Galcanezumab 120 mg/month (with initial loading dose of 240 mg) was administered in patients with high-frequency episodic or chronic migraine for 3 months.

### Classification and definition of responders

**Fig. 2:**
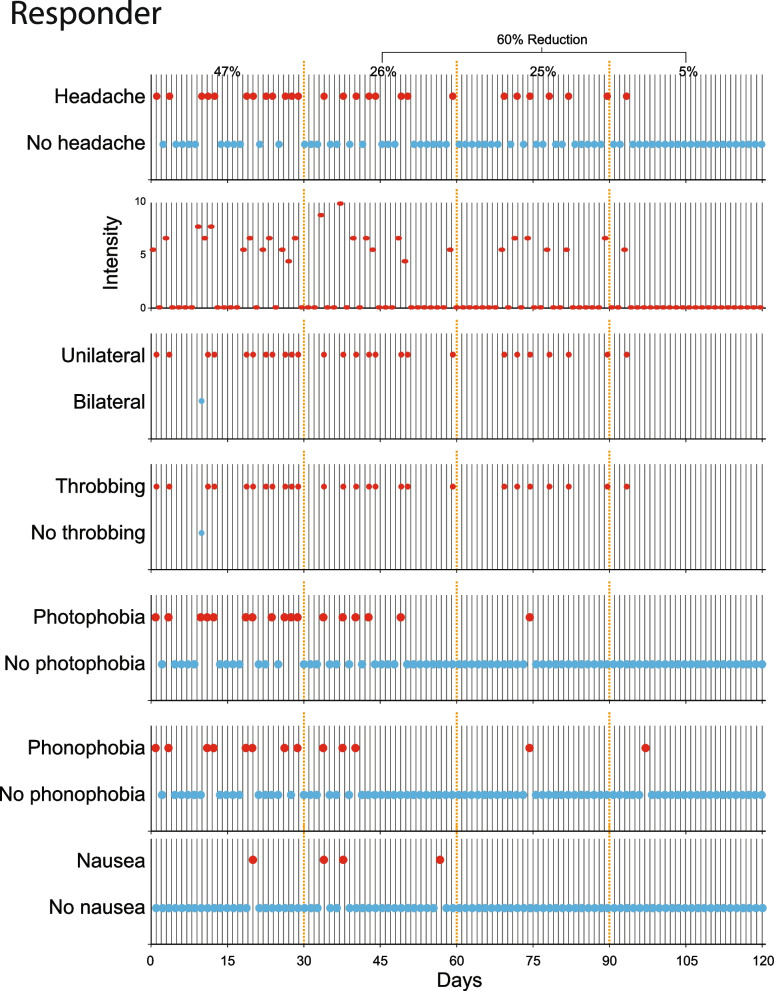
Identification of a galcanezumab responder. Patient’s e-diary entry data were used to compute percentage of monthly migraine and headache days during the 1-month pre-treatment period and the 3-month treatment period. Daily documentation of headache intensity, laterality, throbbing, photophobia, phonophobia, and nausea were used to distinguish between headache and migraine days. Note that the percentage of monthly migraine days (MMD) decreased by > 50% during the treatment period (from 47% before treatment to 18.6% during the 3 months of treatment)

**Fig. 3:**
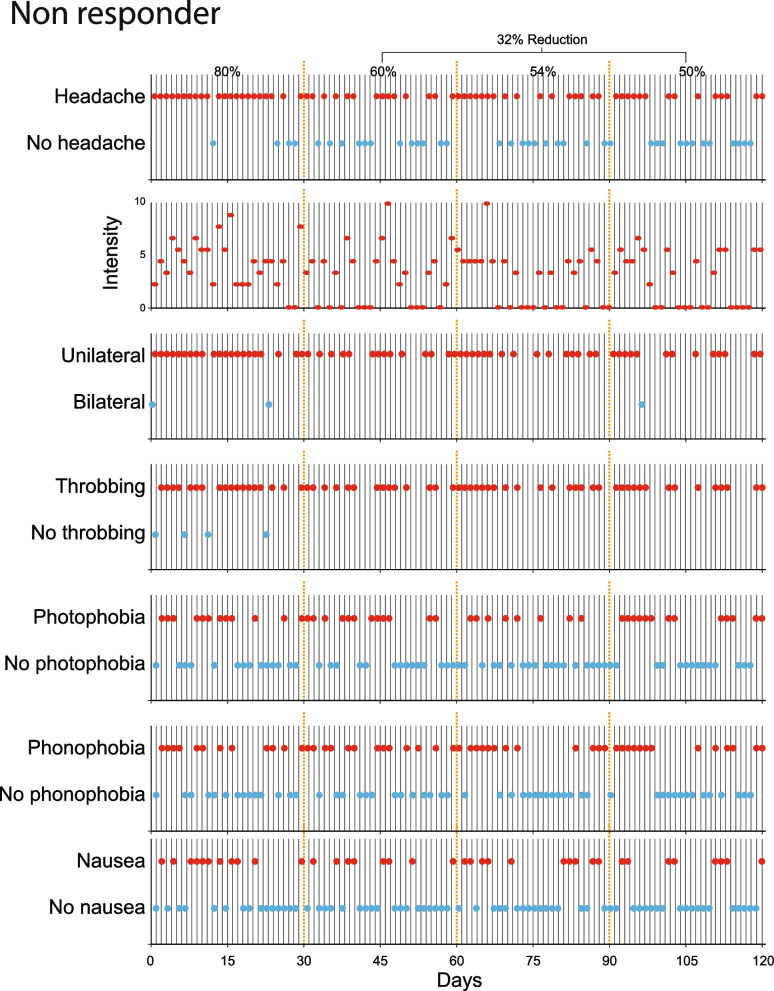
Identification of galcanezumab non-responder. Note that the percentage of monthly migraine days (MMD) decreased by < 50% during the treatment period (from 80% before treatment to 54.6% during the 3 months of treatment)

Patients experiencing 8–14 migraine days per month (MMD) were classified as high-frequency episodic migraine (HFEM) [46]. Those experiencing > 15 MMD were classified as chronic migraine (CM) patients [46]. Galcanezumab responders were patients whose percentage of monthly migraine days decreased by ≥ 50% during the 3-month treatment period (compared to the percentage of migraine days in the 1-month pre-treatment period) (Fig. 2). Conversely, galcanezumab non-responders were patients whose percentage of monthly migraine days decreased by < 50% during the 3-month treatment period (compared to the percentage of migraine days in the 1-month pre-treatment period) (Fig. 3). The 50% change in migraine days per month was adopted as it is the standard in defining responders in phase 3, placebo-controlled studies [47–49]. A priori, we also sought to analyze participants whose migraine days decreased by ≥ 70% during the 3-month treatment period (called here super-responders) and participants whose percentage of migraine days decreased by < 30% during the 3-month treatment period super non-responders (called here super non-responders).

### Statistics

The percentage of migraine days per month, photophobia, phonophobia and nausea, and changes in headache intensity was calculated based on daily entries, and analyzed using descriptive statistics such as means and standard deviations (SD), medians and interquartile ranges, and frequency counts with percentages. To determine whether the data were normally distributed, we used the Shapiro–Wilk test. Since our data were not normally distributed, non-parametric repeated measure test was applied (Friedman test) and post-hoc analysis (Bonferroni) performed. Level of significance was set at 0.05.

The primary outcome of this study was change in occurrence of each premonitory symptom and each trigger identified by participants as appearing before compared to after the treatment period. The resulting occurrences and their descriptive statistics were computed. The differences between those whose premonitory symptoms and triggers were followed by the headache were computed, and the criteria for responders, non-responders, super-responders, and super non-responders were applied. Of all identified premonitory symptoms and triggers, only those whose incidence at baseline was > 20% (i.e., was reported by more than 20% of the participants) were analyzed individually. Those analyzing the incidence of premonitory symptoms and triggers were blinded to the treatment outcome of each participant. The required sample size estimate was obtained to provide sufficient power to evaluate several inferences estimated within two statistical models. The sample size provided 80% to detect paired differences in proportions as small as 17% assuming a two-tailed alpha = 0.05, and a within-person correlation of *r* = 0.75.

### Data and materials availability

The data that support the findings of this study are available from the corresponding author, upon reasonable request.

## Results

### Study sample

Sixty-four CGRP-mAb-naïve episodic and chronic migraine patients were recruited to this study. Forty-six of them completed the 4-month e-diary (1 month before and 3 months during treatment) and did not miss a single treatment cycle. Based on the percent reduction in MMD, 27 (58%) patients were classified as responders ( >50% reduction in MMD), 11 (24%) as super-responders (> 70% reduction in MMD), 19 (42%) as non-responders (< 50% reduction in MMD), and 8 (17%) as super non-responders (< 30% reduction in MMD). Their demographics, classification, and percent reduction in MMD are shown in Tables 1 and 2. Demographics summary and comparisons between the 27 responders and 19 non-responders is provided in Table 3. The responders and non-responders did not differ in any of their demographic values, whereas super non-responders were 12 years older than super responders (p = 0.042), and they had experienced migraine 13 more years (*p* = 0.047).

**Table 1:** Responders demographics, classification and reduction in migraine days per month

	Demographics						% of migraine days per month				Response
	Age	Years w/migraine	Gender	BMI	MIDAS	Classification	Baseline	1 month	2 months	3 months	% reduction (3 months vs baseline)
Patient 1	22	11	Female	37.6	moderate	EM (HF)	32.1	10.0	9.7	8.0	71.3
Patient 2	32	11	Female	23.9	severe	EM (LF)	26.2	13.3	6.5	6.3	66.9
Patient 3	28	20	Male	21.2	moderate	EM (LF)	25.6	12.5	15.4	0.0	63.7
Patient 4	34	7	Female	36	severe	EM (HF)	42.4	13.0	4.3	37.5	56.9
Patient 5	54	9	Female	29.8	severe	EM (HF)	45.5	33.3	12.5	20.0	51.7
Patient 6	27	3	Female	26.5	severe	EM (HF)	48.6	52.6	5.3	14.7	50.2
Patient 7	40	21	Male	25.1	severe	EM (HF)	47.1	26.1	25.0	5.3	60.1
Patient 8	38	26	Female	26.7	moderate	EM (HF)	31.1	4.0	0.0	0.0	95.7
Patient 9	30	13	Male	19.2	moderate	EM (HF)	28.0	8.5	16.7	2.6	66.9
Patient 10	46	35	Male	28.4	severe	EM (HF)	39.3	37.5	20.0	0.0	51.2
Patient 11	49	34	Female	22.9	moderate	EM (HF)	47.1	18.5	7.1	11.1	74.0
Patient 12	27	1	Female	19.5	severe	EM (HF)	39.3	14.3	3.2	6.5	79.7
Patient 13	24	13	Female	24.2	severe	EM (HF)	36.7	30.3	10.3	13.1	51.2
Patient 14	37	22	Female	22.3	severe	EM (HF)	36.8	4.2	14.3	8.3	75.8
Patient 15	42	28	Female	23.8	severe	EM (HF)	41.4	10.3	13.8	20.0	64.4
Patient 16	36	3	Female	25.8	severe	EM (HF)	35.1	9.5	4.8	27.3	60.6
Patient 17	23	5	Female	37	severe	EM (HF)	39.1	20.0	17.9	10.3	58.9
Patient 18	25	13	Female	25	severe	EM (HF)	42.1	16.7	13.3	21.4	59.3
Patient 19	27	20	Male	24.4	severe	EM (HF)	33.3	20.0	9.4	0.0	70.6
Patient 20	25	17	Female	21	severe	CM	83.9	22.7	28.1	17.4	72.9
Patient 21	26	10	Female	23	severe	CM	64.6	7.1	4.2	3.3	92.4
Patient 22	39	15	Female	25.7	severe	CM	50.0	15.4	13.3	6.9	76.3
Patient 23	32	2	Female	25.1	mild	CM	63.6	36.7	35.7	13.3	55.1
Patient 24	34	25	Female	25.5	severe	CM	50.0	11.1	17.6	21.4	66.5
Patient 25	41	20	Female	25.1	severe	CM	57.1	32.3	20.7	17.2	59.1
Patient 26	21	10	Female	18.5	severe	CM	85.7	38.5	8.3	16.7	75.3
Patient 27	26	8	Female	25.9	severe	CM	59.4	9.5	9.5	7.1	85.3

**Table 2:** Non-responders demographics, classification, and reduction in migraine days per month

	Demographics						% of migraine days per month				Response
	Age	Years w/migraine	Gender	BMI	MIDAS	Classification	Baseline	1 month	2 months	3 months	% reduction (3 months vs baseline)
Patient 1	45	21	Female	38.7	little	EM (HF)	44.2	16.1	25.0	50.0	31.3
Patient 2	43	25	Female	40.9	moderate	EM (HF)	38.7	45.5	15.6	50.0	4.3
Patient 3	22	1	Female	22.2	little	EM (LF)	12.9	8.0	8.3	3.4	48.9
Patient 4	40	31	Female	23.5	severe	EM (HF)	32.5	37.5	22.6	20.7	17.2
Patient 5	22	8	Female	26.6	severe	EM (LF)	13.8	19.4	6.7	4.5	26.1
Patient 6	37	31	Female	26.6	severe	EM (HF)	46.9	26.7	23.8	32.1	41.3
Patient 7	61	39	Male	17.7	mild	EM (HF)	36.4	13.6	33.3	43.5	17.1
Patient 8	24	15	Male	31.8	little	EM (HF)	26.8	6.7	13.6	25.0	43.7
Patient 9	33	4	Female	24.3	severe	CM	54.3	29.2	50.0	27.1	34.7
Patient 10	30	15	Female	22.1	severe	CM	80.0	59.3	54.2	50.0	31.9
Patient 11	63	21	Male	23.3	mild	CM	51.3	32.0	24.1	23.8	48.0
Patient 12	52	37	Female	20.9	severe	CM	57.8	64.7	40.7	36.0	18.4
Patient 13	26	1	Female	22.8	severe	CM	63.0	40.0	27.6	43.8	41.1
Patient 14	38	25	Female	20	severe	CM	50.0	37.9	39.3	31.8	27.3
Patient 15	22	2	Female	25	severe	CM	56.7	22.0	39.4	33.3	44.3
Patient 16	21	8	Female	20.4	moderate	CM	66.7	45.8	37.5	42.1	37.3
Patient 17	27	16	Female	23.6	moderate	CM	80.6	84.8	64.5	60.0	13.5
Patient 18	35	20	Female	23.2	severe	CM	51.5	31.8	37.9	35.3	32.0
Patient 19	53	45	Female	21.8	severe	CM	100	96.3	85.7	95.7	7.4

**Table 3:** Baseline demographics, classifications, and disease characteristics of all, non-responders, responders, super non-responders and super-responders

		All patients	Non responders	Responders	*P* value	Super non responders	Super responders	*P* value
Age		34.3 (10.9)	36.5 (13.3)	32.7 (8.6)	0.56 (Mann–Whitney)	42 (13.2)	30.6 (8.7)	0.042 (Mann–Whitney)
Sex	Female	38 (82%)	16 (84%)	22 (82%)		7 (88%)	10 (91%)	
	Male	8 (18%)	3 (16%)	5 (18%)	1 (Fisher exact)	1 (12%)	1 (9%)	1 (Fisher exact)
Years with migraine		16.6 (11.2)	19.2 (13.3)	14.8 (9.4)	0.31 (Mann–Whitney)	28.2 (28)	15.8 (9.2)	0.047 (Mann–Whitney)
% migraine days per month		47.7 (18.4)	50.7 (22.0)	45.5 (15.4)	0.28 (Mann–Whitney)	51.2 (27.8)	51.2 (19.9)	0.93 (Mann–Whitney)
Classification	EM	27 (58%)	8 (42%)	19 (70%)		4 (50%)	6 (54%)	
	CM	19 (42%)	11 (58%)	8 (30%)	0.072 (Fisher exact)	4 (50%)	5 (46%)	1 (Fisher exact)
BMI		25.3 (5.3)	25.0 (6.0)	25.5 (4.8)	0.25 (Mann–Whitney)	24.3 (7.1)	24.3 (5.1)	(Mann–Whitney)
MIDAS	little	3 (7%)	3 (15%)	0		0	0	
	mild	3 (7%)	2 (10%)	1 (4%)		1 (12%)	0	
	moderate	8 (17%)	3 (15%)	5 (18%)		2 (25%)	3 (27%)	
	severe	32 (69%)	11 (60%)	21 (78%)	0.13 (Fisher exact)	5 (63%)	8 (73%)	0.77 (Fisher exact)
Aura (visual, sensory and speech)		28 (60%)	13 (68%)	15 (55%)	0.54 (Mann–Whitney)	4 (50%)	5 (45%)	1 (Mann–Whitney)

### Galcanezumab effects on migraine frequency and associated symptoms

#### Migraine days per month

**Fig. 4:**
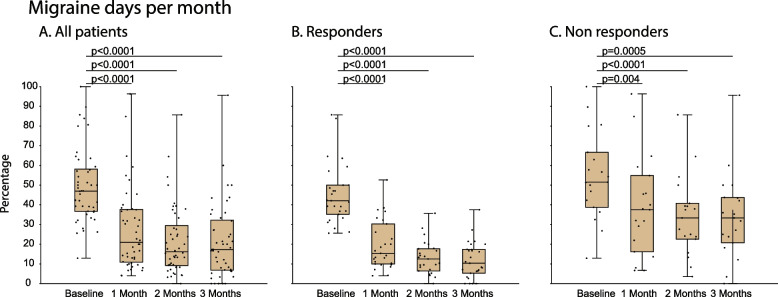
Galcanezumab effects on migraine days per month in (**A**) all 46 patients, (**B**) 24 responders only, and (**C**) 19 non-responders. Differences in percentages of migraine days per month at baseline (before treatment) and in each of the 3 treatment months are shown in box-and-whisker plots (median [IQR]) combined with scatterplots of individual values. Note that the percentage of migraine days per month decreased significantly, although to a different extent, in all 3 groups

In all 46 patients (i.e., in the undivided population), the percentage of MMD was 46.9% [36.6–58.1] (median [IQR]) at baseline, 20.9% [10.9–37.6] after one month of treatment, 16.1% [9.1–29.4] after two, and 17.3% [6.7–32.1] after three (χ^2^ = 76.08, DF = 3, *p* < 0.0001, Friedman test). The post hoc analysis (Bonferroni) yielded significant reduction for all 3 months compared to baseline (Fig. 4A). In the 27 responders, the percentage of MMD was 42.1% [35.1–50.0] at baseline, 15.3% [10.0–30.3] after one, 12.5% [6.4–17] after two, and 10.3% [5.2–17.3] after three months of treatment (χ^2^ = 54.1, DF = 3, *p* < 0.0001, Friedman test). The post hoc analysis (Bonferroni) yielded significant reduction for all 3 months compared to baseline (Fig. 4B). In the 19 non-responders, the percentage of MMD was 51.5% [38.7–66.6] at baseline, 37.5% [16.1–54.8] after one, 33.3% [22.5–40.7] after two, and 33.3% [20.6–43.8] after three months of treatment (χ^2^ = 23.2, DF = 3, *p* < 0.0001, Friedman test). The post hoc analysis (Bonferroni) yielded significant reduction for all 3 months compared to baseline (Fig. 4C).

#### Headache intensity (during migraine days)

**Fig. 5:**
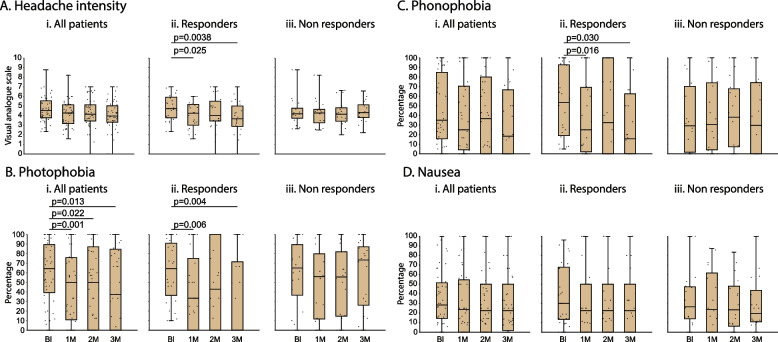
Headache intensity (**A**) and incidences of photophobia (**B**), phonophobia (**C**), and nausea (**D**) during migraine attacks. Box-and-whisker plots (median [IQR]) combined with scatterplots of individual values are illustrated for each of the 4 periods (baseline and during the 3 months treatment). Note that in spite of the decrease in migraine days per month (Fig. 4), the remaining post-treatment attacks were mostly migraineous as indicated by the headache intensity and continuous presence of photophobia, phonophobia and nausea. Abbreviations: Bl - baseline, 1M - 1 month, 2M - 2 month, 3M - 3 month

In all 46 patients, headache intensity (VAS 0–10) was 4.5 [3.7–5.5] at baseline, 4.2 [3.1–5.1] after one, 4.1 [3.4–5.1] after two, and 3.9 [3.3–5.0] after three months of treatment (χ^2^ = 4.6, DF = 3, *p* = 0.19, Friedman test). (Fig. 5Ai). In the 27 responders, headache intensity was 4.71 [3.7–5.9] at baseline, 4.2 [3.0–5.1] after one, 4.0 [3.4–5.5] after two, and 3.6 [2.8–5.0] after three months of treatment (χ^2^ = 9.6, DF = 3, p = 0.02, Friedman test). The post hoc analysis (Bonferroni) yielded a significant difference for months 1 and 3 compared to baseline (Fig. 5Aii). In the 19 non-responders, headache intensity was 4.1 [3.7–4.7] at baseline, 4.2 [3.2–4.6] after one, 4.1 [3.4–4.8] after two, and 4.3 [3.8–5.1] after three months of treatment (χ^2^ = 1.67, DF = 3, p = 0.65, Friedman test) (Fig. 5Aiii).

#### Photophobia (during migraine days)

In all 46 patients, the incidence of photophobia was 64.2 [39.3–89.3] (median [IQR]) at baseline, 50.0 [10.7–75.7] after one, 50.0 [0–87.1] after two, and 37.5 [0–84.5] after three months of treatment (χ^2^ = 12.5, DF = 3, *p* = 0.001, Friedman test). The post hoc analysis (Bonferroni) yielded significant reduction for all 3 months compared to baseline (Fig. 5Bi). In the 27 responders, the incidence of photophobia was 64.2 [36.3–90.9] at baseline, 33.3 [0–75.0] after one, 42.8 [0–100] after two, and 0 [0–71.4] after three months of treatment (χ^2^ = 8.77, DF = 3, *p* = 0.01, Friedman test). The post hoc analysis (Bonferroni) yielded significant reduction for months 1 and 3 compared to baseline (Fig. 5Bii). In the 19 non-responders, the incidence of photophobia was 64.9 [36.4–89.3] at baseline, 55.9 [11.6–79.8] after one, 55.4 [14.6–81.8] after two, and 73.2 [26.0–87.1] after three months of treatment (χ^2^ = 6.68, DF = 3, *p* = 0.065, Friedman test) (Fig. 5Biii).

#### Phonophobia (during migraine days)

In all 46 patients, the incidence of phonophobia was 34.9 [15.5–84.7] (median [IQR]) at baseline, 25.0 [4.1–70.4] after one, 36.6 [0–80.1] after two, and 18.3 [0–66.6] after three months of treatment (χ^2^ = 4.95, DF = 3, *p* = 0.12, Friedman test) (Fig. 5Ci). In the 27 responders, the incidence of phonophobia was 53.5 [18.7–92.7] at baseline, 25.0 [2.0–69.1] after one, 32.5 [0–100] after two, and 15.4 [0–62.5] after three months of treatment (χ^2^ = 7.46, DF = 3, *p* = 0.034, Friedman test). The post hoc analysis (Bonferroni) yielded significant reduction for months 1 and 3 compared to baseline (Fig. 5Cii). In the 19 non-responders, the incidence of phonophobia was 29.1 [1.5–70.2] at baseline, 30.6 [4.1–73.8] after one, 38.1 [7.1–67.8] after two, and 29.5 [0–74.1] after three months of treatment (χ^2^ = 1.93, DF = 3, *p* = 0.52, Friedman test) (Fig. 5Ciii).

#### Nausea (during migraine days)

In all 46 patients, the incidence of nausea was 28.3 [14.2–51.4] (median [IQR]) at baseline, 23.6 [0–54.6] after one, 22.5 [0–50.0] after two, and 22.5 [1.8–50.0] after three months of treatment (χ^2^ = 1.20, DF = 3, *p* = 0.72, Friedman test) (Fig. 5Di). In the 27 responders, the incidence of nausea was 30.0 [13.2–67.8] at baseline, 22.5 [0–50.0] after one, 22.5 [0–50.0] after two, and 22.5 [0–50.0] after three months of treatment (χ^2^ = 1.60, DF = 3, *p* = 0.62, Friedman test) (Fig. 5Dii). In the 19 non-responders, the incidence of nausea was 26.3 [13.9–47.2] at baseline, 23.6 [0–61.7] after one, 23.3 [6.2–48.0] after two, and 19.3 [10.8–43.6] after three months of treatment (χ^2^ = 0.85, DF = 3, *p* = 083, Friedman test) (Fig. 5Diii).

### Premonitory symptoms

#### Incidence of premonitory symptoms that were followed by headache

Of the many different premonitory symptoms identified by all participants at the pre-treatment interview, 12 were considered for further analysis based on their incidence **–** i.e., reported by > 10% of the patients (Table 4). Of these, 9 premonitory symptoms were reported by > 20%, and 3 by < 20% (Table 4). Only the 9 most reported premonitory symptoms were analyzed individually before and after treatment in responders and non-responders. Of note, at the pre-treatment interview, the occurrence of all but 2 premonitory symptoms was similar in responders and non-responders (*p* > 0.05, DF = 1, Fisher Exact). Only phonophobia and yawning differed: they were significantly higher in responders than non-responders (*p* ≤ 0.04, DF = 1, Fisher Exact).

**Table 4:** Incidence of premonitory symptoms that were followed by headache prior to galcanezumab treatment

	**Incidence %**			
**Premonitory symptoms**	**All patients**	**Responders**	**Non responders**	**Fisher exact**
Neck stiffness	54.3	59.3	47.4	0.550
Cognitive impairment	54.3	63.0	42.1	0.231
Photophobia	39.1	48.1	26.3	0.21
Irritability	39.1	48.1	26.3	0.219
Fatigue	37.0	44.4	26.3	0.235
Stress/nervous	32.6	33.3	31.6	1.000
Vertigo/dizziness	30.4	37.0	21.1	0.335
Nausea	28.3	29.6	26.3	1.000
Phonophobia	28.3	40.7	10.5	0.044
Osmophobia	17.4	22.2	10.5	0.439
Ringing in the ear	15.2	18.5	10.5	0.682
Yawning	13.0	22.2	0.0	0.034

#### Galcanezumab effects on incidence of premonitory symptoms that were followed by headache in responders vs. non-responderss

**Fig. 6:**
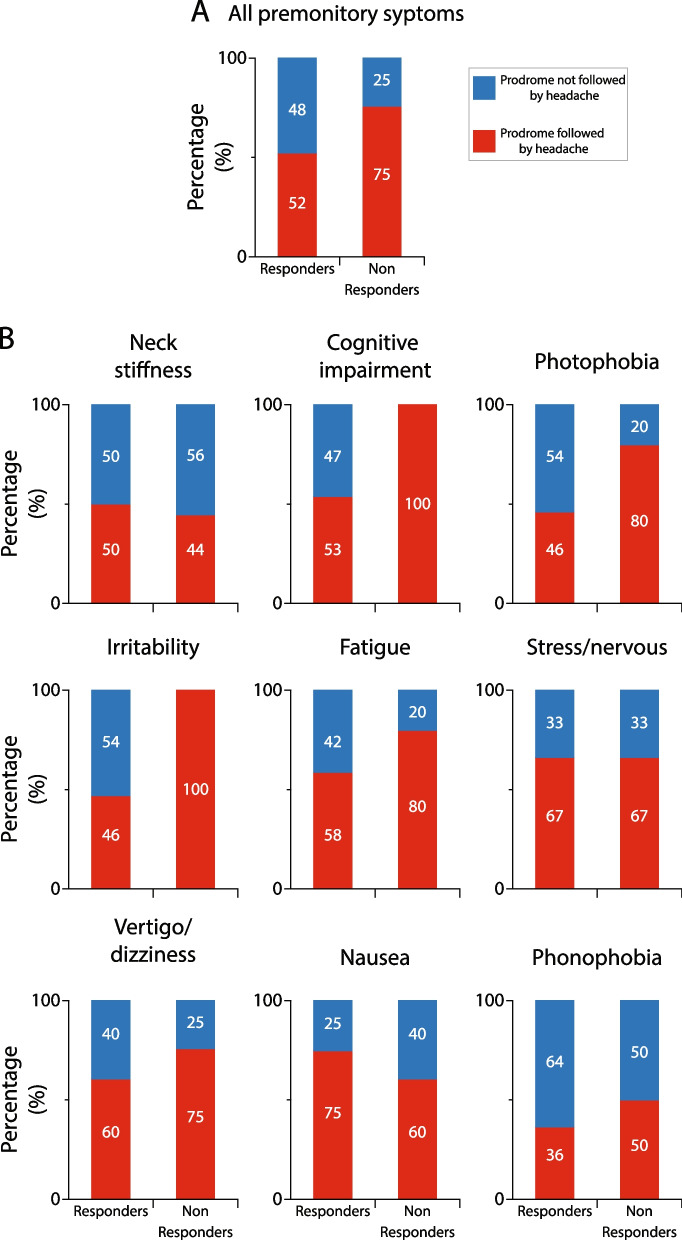
Galcanezumab effects on incidence of headache after occurrence of premonitory symptoms in responders and non-responders. **A** Incidence of all premonitory symptoms that were followed by headache decrease by the galcanezumab treatment in both, responders and non-responders. Note that after occurrence of premonitory symptoms, responders experienced significantly less headaches than non-responders (*p* = 0.004, DF = 1, Fisher exact). **B** Incidences of headache following occurrence of each of the 9 most common premonitory symptoms (i.e., premonitory symptoms that prior to treatment were followed by headache in > 20% of all patients) during the 3-month treatment period. The 100% (in **A** and **B**) represents incidences of all (**A**) and individual (**B**) premonitory symptoms that were followed by headache prior to treatment initiation

Given the large variability in incidence of premonitory symptoms within and between patients, the study was powered enough to allow comparisons between incidences of all premonitory symptoms in all responders vs. all premonitory symptoms in all non-responders, but not between incidences of individual premonitory symptoms. After treatment with galcanezumab, the incidence of all premonitory symptoms that were followed by headache decreased by 48% in the responders vs. 25% in the non-responders (*p* = 0.004, DF = 1, Fisher exact) (Fig. 6A). Descriptive analyses of the incidence of each of the 9 most common premonitory symptoms that were followed by headache after galcanezumab treatment in the responders and non-responders is shown in Fig. 6B. After treatment with galcanezumab, the incidence of headache following occurrence of 7 premonitory symptoms was reduced in the responders and non-responders, whereas the incidence of headache after occurrence of 2 premonitory symptoms (irritability, cognitive impairment) was reduced only in the responders.

#### Galcanezumab effects on incidence of premonitory symptoms that were followed by headache in super-responders vs. super non-responders

**Fig. 7:**
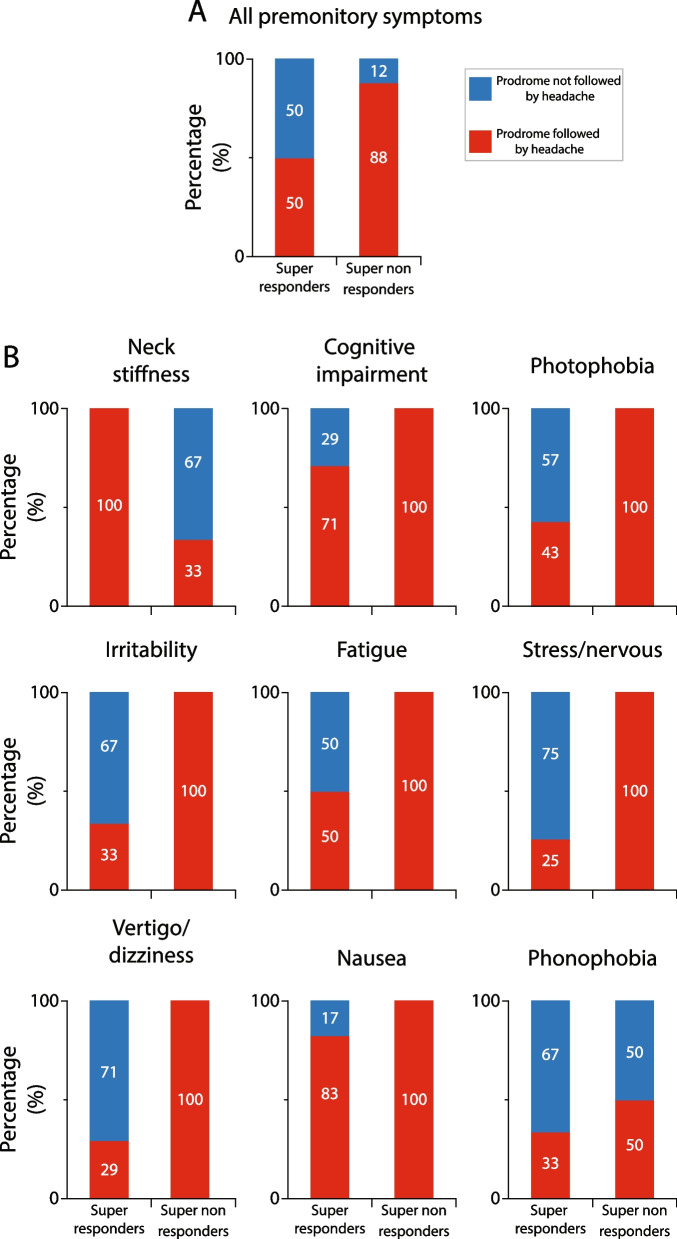
Galcanezumab effects on incidence of headache after occurrence of premonitory symptoms in super-responders and super non-responders. **A** Incidence of all premonitory symptoms that were followed by headache decrease nearly fourfold more in the super-responders than the super non-responders. Note that after occurrence of premonitory symptoms, super-responders experienced significantly less headaches than super non-responders (*p* = 0.001, DF = 1, Fisher exact). **B** Incidences of headache following occurrence of each of the 9 most common premonitory symptoms during the 3-month treatment period. Note that in the super non-responder group galcanezumab treatment did not reduce incidence of headache after occurrence of 7/9 premonitory symptoms

After treatment with galcanezumab, the incidence of all premonitory symptoms that were followed by headache decreased by 50% in the super-responders vs. 12% in the super non-responders (*p* = 0.001, DF = 1, Fisher exact) (Fig. 7A). Descriptive analyses of the incidence of each of the 9 most common premonitory symptoms that were followed by headache after galcanezumab treatment in the responders and non-responders is shown in Fig. 7B. In contrast to the finding in the 50% responders, after treatment with galcanezumab, the incidence of headache following occurrence of 7 premonitory symptoms was reduced only in the super-responders, whereas the incidence of headache after occurrence of phonophobia was reduced in both groups, and after occurrence of neck stiffness it was reduced only in the super non-responders.

### Aura

#### Galcanezumab effects on incidence of auras that were followed by headache in responders, super-responders, non-responders, and super non-responders

**Fig. 8:**
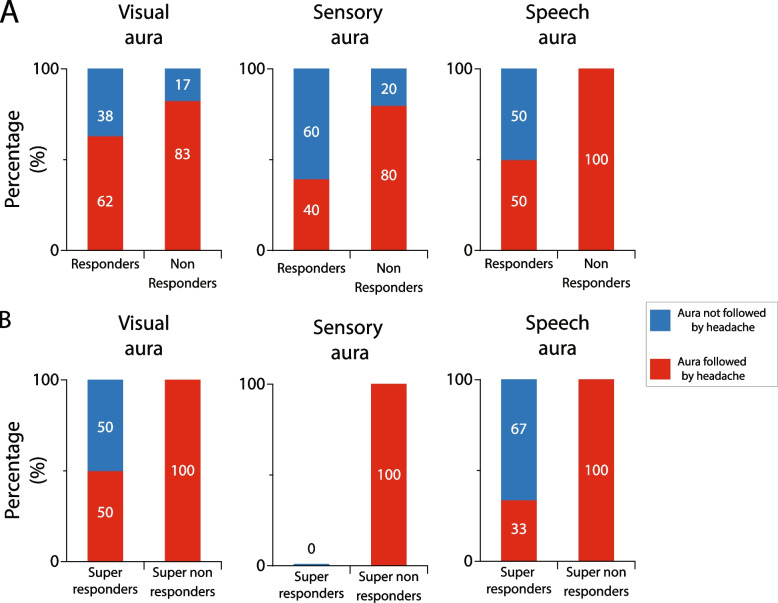
Galcanezumab effects on incidence of headache after occurrence of aura in responders vs. non-responders (**A**), and super-responders vs. super non-responders (**B**). Complete absence of effect in the super non-responders suggest the galcanezumab ability to reduce incidence of headache after aura is secondary to the reduction in migraine days per month

The overall percentage of participants diagnosed with migraine aura was 30.4. This include participants experiencing any one of the classical symptoms of visual aura only, sensory aura only, speech aura only (as defined in Table 5), or a combination of visual and sensory or visual and speech aura (Table 5). These incidences were similar in responders (visual 29.6%, sensory 18.5%, speech 7.4%) and non-responders (visual 31.6%, sensory 26.3%, speech 10.5%) (*p* > 0.05, DF = 1, Fisher Exact). After treatment with galcanezumab, the commencement of headache after occurrence of visual and sensory aura was reduced in responders and non-responders, whereas after speech aura, headache incidence was reduced only in the responders (Fig. 8A). In contrast, the incidence of headache following visual, sensory, and speech aura was reduced in the super-responders but not in the super non-responders (Fig. 8B).

**Table 5:** Incidences of auras that were followed by headache prior to galcanezumab treatment

	**Incidence %**			
**AURA**	**All patients**	**Responders**	**Non responders**	**Fisher exact**
visual aura^a^	30.4	29.6	31.6	1.000
sensory aura^b^	21.7	18.5	26.3	0.718
speech aura^c^	8.7	7.4	10.5	1.000

^a^visual aura was determined when participants described experiencing zigzag lines, scintillation, scintillating scotoma, hemianopsia, fortification spectra, flickering lights, flashes of bright light, blind spots, c-shaped zigzag lines – All at least 20 min before onset of headache

^b^Sensory aura was determined when participants described experiencing tingling, numbness, or paresthesia in their fingers, hands, face, or legs

^c^Speech aura was determined when participants described having trouble with word-finding difficulty or speech production

### Triggers

#### Incidence of triggers that were followed by headache at baseline

Eleven triggers were reported by > 20% of all participants (Table 6) and were thus analyzed individually before and after treatment with galcanezumab in responders vs. non-responders. Of note, at the pre-treatment interview, the incidence of all 11 triggers was similar in responders and non-responders (*p* > 0.05, DF = 1, Fisher Exact).

**Table 6:** Incidences of triggers that initiated migraine headache before treatment

	**Incidence %**			
**Triggers**	**All patients**	**Responders**	**Non responders**	**Fisher exact**
Sleep little/much	76.1	66.7	89.5	0.092
Skip meal	63.0	59.3	68.4	0.554
Stress	58.7	59.3	57.9	1.000
Bright light	58.7	55.6	63.2	0.762
Let down^a^	56.5	48.1	68.4	0.231
Not drinking	56.5	51.9	63.2	0.550
Weather	54.3	44.4	68.4	0.139
Menses	52.2	48.1	57.9	0.561
Food/wine	45.7	44.4	47.4	1.000
Smell	37.0	29.6	47.4	0.354
Too hot	21.7	14.8	31.6	0.277

^a^Let down after stress

#### Galcanezumab effects on incidence of triggers that were followed by headache in responders vs. non-responders

**Fig. 9:**
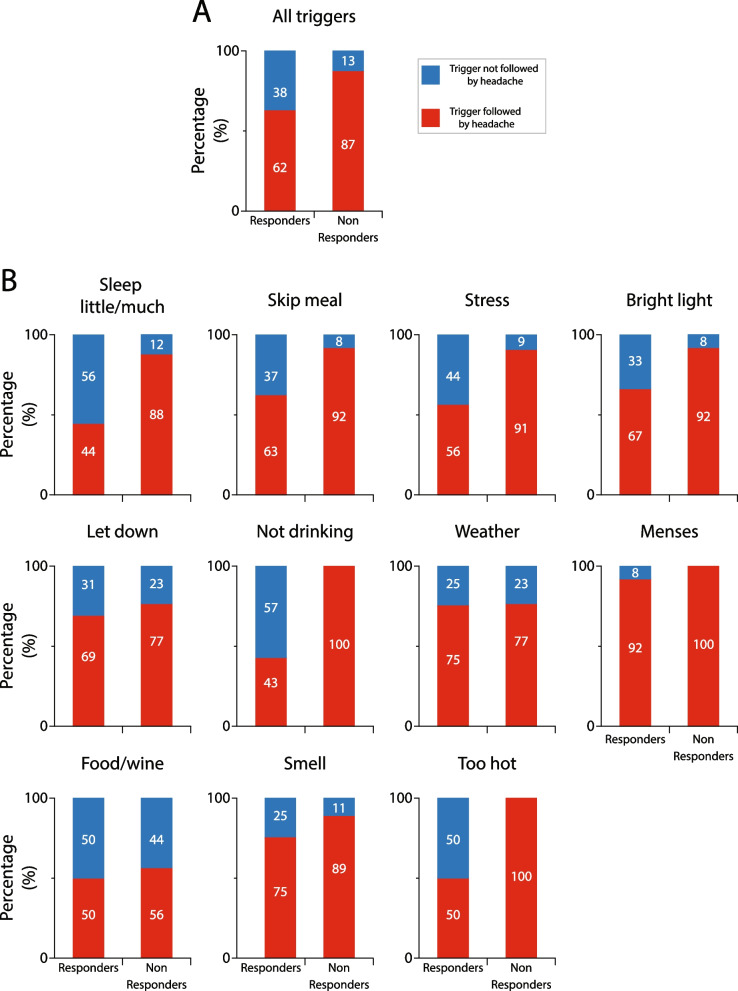
Galcanezumab effects on incidence of triggers that were followed by headache in responders and non-responders. **A** Incidence of all triggers that were followed by headache after galcanezumab treatment decreased in both, responders and non-responders. Note that after occurrence of, or exposure to triggers, responders experienced significantly less headaches than non-responders (*p* = 0.0001, DF = 1, Fisher exact). **B** Incidences of headache following occurrence of, or exposure to each trigger during the 3-month treatment period. The 100% (in **A** and **B**) represents incidences of all (**A**) and individual (**B**) premonitory symptoms that were followed by headache prior to treatment initiation

Given the large variability in incidence of triggers within and between patients, the study was powered enough to allow comparisons between incidences of all triggers in all responders vs. all triggers in all non-responders, but not between incidences of individual triggers. The number of triggers that were collectively reported by all responders and all non-responders to induce headache before treatment dropped by 38 and 13%, respectively, after treatment (p < 0.0001, DF = 1, Fisher exact) (Fig. 9A). Descriptive analyses of the incidence of each of the 11 most common triggers that were followed by headache after galcanezumab treatment in the responders and non-responders is shown in Fig. 9B. After treatment with galcanezumab, the number of headaches that followed 8 triggers was reduced in both the responders and non-responders, whereas the number of headaches that followed 3 triggers (not drinking, menses and being too hot) was reduced only in the responders.

#### Galcanezumab effects on incidence of triggers that were followed by headache in super-responders vs. super non-responders

**Fig. 10:**
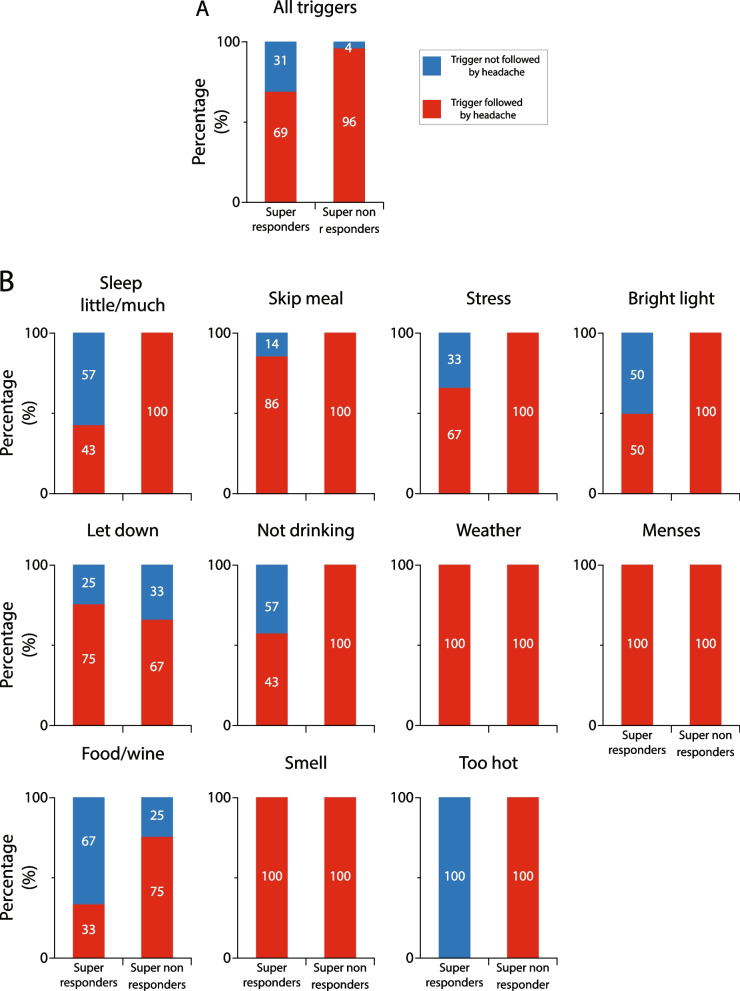
Galcanezumab effects on incidence of triggers that were followed by headache in super-responders and super non-responders. **A** Incidence of all triggers that were followed by headache after galcanezumab treatment in super-responders and super non-responders. Note that after occurrence of, or exposure to triggers, super-responders experienced significantly less headaches than super non-responders (*p* = 0.0001, DF = 1, Fisher exact). **B** Incidences of headache following occurrence of, or exposure to each trigger during the 3-month treatment period. Note that in the super non-responders, galcanezumab treatment had no effect on 9/11 triggers, and in the super-responders, it had no effect 3/11 triggers

The number of triggers that were collectively reported by all super-responders and all super non-responders to induce headache before treatment dropped by 31 and 4%, respectively, after treatment (*p* = 0.0004, DF = 1, Fisher exact) (Fig. 10A). Descriptive analyses of the incidence of each of the 11 most common triggers that induced headache after galcanezumab treatment in the responders and non-responders is shown in Fig. 10B. In contrast to the finding in the 50% responders, after treatment with galcanezumab, the number of headaches that followed 6 triggers was reduced only in the super-responders, whereas the number of headaches that followed 2 triggers (letdown and food/wine) was reduced in both groups. Remarkably, 3 triggers (weather, odors, and menses) were not affected by the galcanezumab treatment in either group.

## Discussion

We demonstrate that after treatment with galcanezumab, (a) the incidence of premonitory symptoms that were followed by headache decreased by 48% in responders vs. 28% in non-responders, and by 50% in super-responders vs. 12% in super non-responders; (b) the incidence of visual and sensory auras that were followed by headache was reduced in responders, non-responders, and super-responders, but not in super non-responders; (c) the number of triggers followed by headache decreased by 38% in responders vs. 13% in non-responders, and by 31% in super-responders vs. 4% in super non-responders; (d) the incidence of headache following occurrence of some premonitory symptoms (i.e., cognitive impairment, irritability) was reduced in responders and super-responders but not in non-responders, whereas incidence of headache after occurrence of other premonitory symptoms (e.g., fatigue, nausea, and dizziness/vertigo) was reduced in responders, non-responders, and super-responders, but not in super non-responders; (e) the incidence of headache following exposure to some triggers (i.e., not drinking enough, being too hot) was reduced in responders and super-responders but not in non-responders, whereas incidence of headache after exposure to other triggers (i.e., stress, sleeping too little, bright light) was reduced in responders, non-responders, and super-responders, but not in super non-responders; (f) the incidence of headache around the first day of menses was not reduced in either group; and (g) the incidence of headache after occurrence of visual, sensory, or speech aura was reduced in all (with one exception) but the super non-responder group. Mechanistically, these findings suggest that even a mild decrease in MMD (30–50%) is sufficient to partially reverse pain impact on the excitability and responsivity of neurons involved in the generation of certain premonitory symptoms and triggers of migraine. Clinically, these findings provide further justification for lowering the definition threshold of responders from > 50 to > 30% reduction in MMD.

### Responders vs. non-responders

Our working hypothesis was that common premonitory symptoms (e.g., feeling irritated, tired, nauseous, neck pain) and triggers (e.g., not sleeping enough, skipping meals and feeling hungry, being exposed to bright light or strong odors) lead to the initiation of headache in people with migraine but not in people without migraine. This is because the frequent invasion of multiple brain areas with nociceptive signals that originate in the meninges during migraine alters the physiological properties of hypothalamic, cortical, subcortical and brainstem neurons involved in maintenance of homeostasis and regulation of sleep, body temperature, energy, and hormonal secretion, adjustments to strong visual, auditory and olfactory stimuli, cognitive performance, generation of affect, autonomic responses and the perception of pain. According to this hypothesis, we predicted that the incidence of headache following occurrence of premonitory symptoms and triggers will decrease in patients in whom migraine frequency is reduced by galcanezumab, but not in patients in whom migraine frequency is not reduced by this treatment. Against our prediction, the incidence of headache following occurrence of premonitory symptoms and triggers decreased (though to a larger extent in responders than non-responders) in both responders and non-responders. Theoretically, there are two ways to interpret these findings. The first is to consider the possibility that galcanezumab’s effects on premonitory symptoms and triggers are secondary to this drug’s ability to cross the blood brain barrier and alter CGRP signaling in the many brain areas involved in the generation of the premonitory symptoms and triggers that were reduced in the non-responders (i.e., that treatment effect is independent of galcanezumab’s ability to reduce migraine frequency). The second is the possibility that the non-responder group included patients whose MMD was reduced by 30–50% and that over time, even such ‘mild’ reduction is sufficient to reduce neuronal hyperexcitability in all brain areas that directly and indirectly receive trigeminovascular input. The findings in the super-responders and super non-responders support the second interpretation.

### Super-responders vs. super non-responders

In patients in whom MMD was reduced by < 30%, 7 of 9 premonitory symptoms (cognitive impairment, photophobia, irritability, fatigue, stress/nervous, dizziness/vertigo, nausea) were completely unaffected by the galcanezumab treatment. In contrast, in patients in which MMD decreased by ≥ 70%, galcanezumab treatment decreased the occurrence of headache that was preceded by all premonitory symptoms but one (neck stiffness). Similarly, the occurrence of headache after exposure to 6 triggers (sleeping too little, skipping meals, stress, bright light, not drinking enough, being too hot) was reduced in the super-responders only. These findings allow us to evaluate the impact of pain more reliably. Based on these data, we propose that when migraine frequency decreases, neurons involved in the generation of premonitory symptoms and triggers use the extended pain-free time to restore normal membrane potential, and consequently their hyperexcitable, hypersensitive, and hyperresponsive state is reversed. Along this line, it is also important to note that super non-responders had experienced migraine attacks for significantly more years than super-responders. This difference further supports the view that there is an accumulative effect on the brain of repeated exposure to the pain of headache, and that effort to prevent migraine should start early (before patients reach the 15 migraine days per month).

### Premonitory symptoms

Mechanistically, it is interesting to note that in all non-responders, cognitive impairment and irritability were completely unaffected by the galcanezumab treatment, and that, in addition, photophobia, fatigue, nausea, dizziness, and feeling stressed and anxious were completely unaffected in the super non-responders. While some of these neurological symptoms could be assigned to particular brain areas (e.g., nausea and area postrema, photophobia and the visual cortex), the majority are ‘generated’ by multiple cortical, subcortical, hypothalamic, thalamic, and brainstem nuclei, and, as such, it may not be possible to assign them with certainty to one area or nucleus [50]. Following principles of neural science, we propose that these premonitory symptoms are generated by neurons and neural circuits whose excitability and responsivity are altered by nociceptive signals that originate in the meninges over time. This proposal does not imply that the premonitory symptoms are initiated by the headache itself. Rather, it suggests that prolonged exposure to pain alters the brain (i.e., the physiological properties of neurons affected by the pain) over time. Such scenario can also explain occurrence of non-headache symptoms outside the headache phase of migraine [51]. In contrast, the finding that the incidence of headache after occurrence of neck stiffness/pain was reduced in the non-responders and super non-responders supports the conclusion that neck pain may not be considered a classical premonitory symptom [52] and that the neuronal circuits that mediate neck pain may not be affected by the nociceptive signals from the meninges. A reasonable speculation is that galcanezumab reduces neck pain by neutralizing CGRP at peripheral sites in deep neck structures.

### Triggers

In principle, a similar argument could be proposed for triggers that were reduced by the galcanezumab treatment in the responders and super-responders but remain completely unaffected by the galcanezumab treatment of the super non-responders. These include predominantly hypothalamic-mediated triggers such as sleeping too little, skipping meals, not drinking enough, and being too hot, and triggers involving cortical and subcortical limbic areas. Mechanistically, it is also important to note that in all 4 groups of patients, 3 triggers (menses, weather changes, exposure to certain odors) were minimally or completely unaffected by the galcanezumab treatment, suggesting that pain signals do not reach brain areas involved in the genesis of these triggers or are unable to alter the involved networks.

### Triggers, prodromes and symptoms

Scientifically, it is reasonable to suggest that triggers, prodromes and symptoms are mediated/generated by distinct and very different neural networks. While it is most likely that different neurons and neural networks mediate different triggers and prodromes, our suggestion that reduced neuronal hyperexcitability may be the common denominator for the reduction in occurrence of headache after exposure to triggers or after occurrence of premonitory symptoms is justified. To simplify this complicated topic, we opted to focus on light as it can be a trigger, a prodrome or a symptom. When it is a *trigger*, it is commonly described as exposure to light (sometimes bright, sometimes computer screen blue, sometimes flickering) that is followed by the classical headache of migraine within a short period of time. When it is a *prodrome*, it is described as general aversion to light (light that is uncomfortable to look at even though it is not bright, or flickering, or blue) 1–2 h before the onset of headache. When it is a symptom, it is commonly described as exacerbation of headache by light during the ictal phase of a migraine attack. The large amount of data that support the view that the visual cortex of migraine patients is hyperexcitable [9, 53–55] is the foundation of our overall proposal. It creates a common denominator to light as a trigger and light as a prodrome. When the visual cortex is hyperexcitable and hyperresponsive, bright and flickering light (i.e., light that is never comfortable) can give rise to a chain of events that culminate in a headache, and light that is usually comfortable can be perceived as aversive during the prodromal phase (as the stimulus is far weaker). Conversely, when cortical hyperexcitability is reduced, bright and flickering lights lose their power to trigger a headache and light that is otherwise comfortable continues to be comfortable.

On the contrary, fundamental differences exist between prodromal and ictal photophobia. By definition, prodromal photophobia does not make the headache more painful as there is no headache or pain yet, whereas ictal photophobia is exacerbating an already existing headache (i.e., makes it more painful). Mechanistically, ictal photophobia is thought to involve convergence of retinal ganglion cells on thalamic trigeminovascular neurons that project to the primary and secondary visual cortices as well the so-called pain matrix (S1, S2 and the insula) [29, 56], while the aversion to light is thought to reflect abnormal behavior of cortical and hypothalamic neurons involved in the regulation of mood, anger, anxiety and autonomic regulation [57]. In this scenario, light activates the visual cortex to a lesser extent during the prodromal phase than during the ictal phase as in the prodromal phase the visual cortex does not receive additional nociceptive signals from thalamic trigeminovascular neurons in the pulvinar, lateral dorsal and lateral posterior thalamic neurons [29, 56].

By far, the most limiting aspect of any further discussion on these topics must take into consideration the fact that we don’t know how any prodrome or trigger gives rise to the typical pain of migraine headache or how any prodrome or trigger (may be except aura) gives rise to activation of a meningeal nociceptor. An exception to this is the case of aura.

### Galcanezumab mechanism of action

While the findings of this study are unlikely to resolve the debate over peripheral vs. central sites of action of CGRP-mAbs, it is fair to propose that if a CGRP-mAb could cross the blood brain barrier and neutralize CGRP in brain areas involved in the generation of premonitory symptoms or the triggering processes, one would expect to find no difference between galcanezumab responders and galcanezumab non-responders. Given our findings, however, we conclude that galcanezumab’s ability to reduce premonitory symptoms and triggers is secondary to its ability to partially or completely reduce the magnitude (number of action potentials) and duration of nociceptive signals that originate in the meninges and reach the brain over many months.

### Caveats

Our study has several methodological limitations. (a) Given that this was a single-arm, open-label, proof-of-concept study, the percentages of responders, super-responders, non-responders, and super non-responders may have been overestimated. However, the percentages of patients achieving 30, 50 and 70% reduction in MMD is not very far from the percentages reported in previous randomized control studies of chronic and episodic migraine patients with galcanezumab [48, 58–61]. Additionally, it must be noted that this was not an efficacy study design; thus, the findings about percentages of responders vs. non-responders should not be considered as an outcome measure. (b) While occurrence of migraine days, throbbing, photophobia, phonophobia, and nausea were captured in the electronic daily headache diary and are, therefore, considered highly reliable, the occurrence of premonitory symptoms or exposure to triggers and whether they were followed by headache was captured by questionnaires and interviews conducted before and after the treatment period. Although we attempted to minimize this weakness by instructing participants to mark premonitory symptoms and triggers as being followed by headache if they happened at least once during the last 2 months of treatment, and no longer causing (i.e., triggers) the headache or being related (premonitory) to it only if they were not followed by a single headache during that period, it must be taken into consideration that the reliability of such data is not as high as data collected with electronic daily headache diary [7]. Adding to this limitation is the known fact that regardless of treatment, many premonitory symptoms and triggers do not predict with high consistency the onset of the headache phase [1, 4–6, 62, 63]. (c) is a premonitory symptom not followed by headache still a premonitory symptom? When this question is deferred to patients, it is difficult not to consider premonitory symptoms as such. Responders repeatedly stated how surprised they were to experience their usual “warning signs” (e.g., visual aura, difficulty finding words, feeling tired, irritated, etc.) but not have them followed by the headache. While philosophically this is a legitimate question, in real-life it must be decided if a full course of visual aura that is not followed by headache is still a migraine aura or a transient ischemic attack? Before a consensus is reached around this issue, it must be listed as a potential caveat.

## Conclusions

In this observational, open-label, cohort study, a peripherally acting CGRP-mAb (galcanezumab) reduced incidence of headache after occurrence of common premonitory symptoms and triggers in those patients in which it reduced migraine frequency by > 30, > 50 and > 70%, but not by < 30%. These findings suggest that some premonitory symptoms and triggers are secondary to hyperexcitability and hyper-responsiveness that develop in neurons that receive prolonged and continuous nociceptive signals from the meninges, and that even mild attenuation of these pain signals is sufficient to restore the normal sensitivity of neurons and neural circuits that mediate these premonitory symptoms and triggers.
